# Digital Mini-LED Lighting Using Organic Thin-Film Transistors Reaching over 100,000 Nits of Luminance

**DOI:** 10.3390/nano15020141

**Published:** 2025-01-17

**Authors:** Chia-Hung Tsai, Yang-En Wu, Chien-Chi Huang, Li-Yin Chen, Fang-Chung Chen, Hao-Chung Kuo

**Affiliations:** 1 Department of Photonics, College of Electrical and Computer Engineering, National Yang Ming Chiao Tung University, Hsinchu 30010, Taiwan; sppsai.ee12@nycu.edu.tw (C.-H.T.); ivanwu.ee12@nycu.edu.tw (Y.-E.W.); cchuang.ee13@nycu.edu.tw (C.-C.H.); lychen@nycu.edu.tw (L.-Y.C.); 2 Smartkem Ltd., Manchester M9 8GQ, UK; 3 Center for Emergent Functional Matter Science, National Yang Ming Chiao Tung University, Hsinchu 30010, Taiwan; 4 Semiconductor Research Center, Hon Hai Research Institute, Taipei 11492, Taiwan

**Keywords:** full-array local dimming (FALD), mini-LED backlights, digital lighting, organic TFTs

## Abstract

This paper demonstrates the use of organic thin-film transistors (OTFTs) to drive active digital mini light-emitting diode (mini-LED) backlights, aiming to achieve exceptional display performance. Our findings reveal that OTFTs can effectively power mini-LED backlights, reaching brightness levels exceeding 100,000 nits. This approach not only enhances image quality but also improves energy efficiency. OTFTs offer a flexible and lightweight alternative to conventional silicon-based transistors, enabling innovative and versatile display designs. The integration of mini-LED technology with OTFTs produces displays with superior contrast ratios, enhanced color brightness, and lower power consumption. This technological advancement is poised to revolutionize high-dynamic-range (HDR) displays, including those in televisions, smartphones, and wearable devices, where the demand for high brightness and energy efficiency is paramount.

## 1. Introduction

Liquid-crystal display (LCD) technology has become mature after decades of mass production and consistent capital investment in the Far East [1]. Backlighting technology for LCDs has undergone significant advancements, aiming to enhance display quality, increase energy efficiency, and improve overall user experience. The primary types of backlighting used in modern LCDs include cold-cathode fluorescent lamps (CCFLs), light-emitting diodes (LEDs), and the more advanced mini light-emitting diode (mini-LED) and micro-LED technologies [2,3]. Conventional backlight technologies include edge-lit LEDs and full-array LEDs. In edge-lit LEDs, LEDs are placed along the edges of the display, and light is distributed across the screen using light guides. This method allows for slimmer display designs but may result in less uniform brightness [4]. On the other hand, full-array LED technology places LEDs uniformly behind the entire screen. This configuration enables local dimming, where specific screen zones can be dimmed or brightened independently, enhancing contrast and energy efficiency [4].

Mini-LEDs are LEDs with sizes typically ranging from 100 to 500 μm, used primarily as backlight sources in LCD panels [5]. The key advantage of mini-LED backlighting lies in its ability to implement local dimming [6]. Local-dimming mini-LED backlight technology is revolutionizing the display industry by enhancing image quality and energy efficiency [7]. Unlike conventional backlighting methods that uniformly illuminate the entire screen, local dimming enables precise control over individual zones of the display. This capability enables the screen to display deeper blacks and brighter whites simultaneously, vastly improving the contrast ratio and overall image clarity. Such enhancements are particularly beneficial for high-dynamic-range (HDR) content, which features pronounced differences between the brightest and darkest parts of the image [8,9]. This technology employs thousands of tiny LEDs as a backlight source, allowing for precise control over individual zones of the display. This precision leads to improved contrast ratios, deeper blacks, and brighter whites, significantly enhancing the viewing experience compared to traditional backlighting methods. There are two primary driving methods used in the production of these tiny LEDs. The first is full-array local dimming (FALD), primarily using passive-matrix (PM) driving [10,11]. Due to its simplicity and cost-effectiveness, this method is suitable for applications where high resolution and fast response times are less critical. However, FALD systems typically have lower refresh rates and slower response times, rendering them less suitable for high-performance displays. The second method is active-matrix (AM) driving, where each mini-LED is controlled individually using thin-film transistors (TFTs) [12]. AM systems, commonly found in modern LCDs and OLED displays, provide faster response times, higher refresh rates, and better overall display performance [13]. While AM systems are more expensive and complex, they deliver superior image quality, which is ideal for high-end televisions, monitors, and other advanced display applications.

Among conventional TFTs [14] (e.g., amorphous silicon (a-Si) TFTs [15], low-temperature polysilicon (LTPS) TFTs [16,17], oxide TFTs [18,19]), organic TFTs (OTFTs) [20,21,22,23] are emerging as a promising technology for flexible and lightweight displays. This study aims to demonstrate the feasibility of using OTFTs for driving active-local-dimming mini-LED backlighting systems. Our results show that the proposed system can achieve a luminance of over 100,000 nits with excellent stability, positioning OTFTs as a promising solution for high-performance displays [24,25].

## 2. Experiment and Fabrication Process

The experimental procedure for integrating mini-LEDs with OTFT backplanes involves three main steps: OTFT backplane fabrication [26], mini-LED die bonding, and powering up the mini-LED backlights.

### 2.1. OTFTs Backplane Fabrication

**Figure 1:**
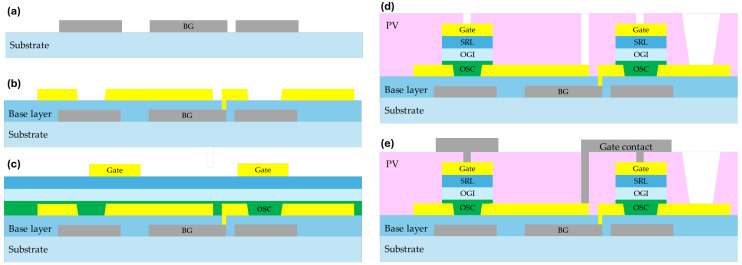
Process flow of OTFT backplane. (**a**) Mask 1: bottom gate metal patterning. (**b**) Mask 2: source and drain metal patterning. (**c**) Mask 3: gate metal patterning. (**d**) Mask 4: passivation via hole patterning. (**e**) Mask 5: gate contact metal patterning.

The fabrication of the solution-processed OTFTs, as shown in Figure 1a–e, followed a 5-mask process to construct AM circuitry and bonding pads for the mini-LEDs.

The process began with the preparation of Corning Eagle XG glass substrates (Corning, AZ, USA), which were cleaned via sonication in a 1% deconex solution at 50 °C for 1 h. This step was followed by rinsing with deionized (DI) water, drying with an air gun, and baking at 70 °C for 60 min to ensure complete removal of contaminants and moisture. As shown in Figure 1a, a Mo/Al/Mo tri-layer was deposited by sputtering to create the back-gate layer. This layer was then patterned using photolithography and subsequently wet-etched with a solution containing phosphoric, acetic, and nitric acids. A buffer layer (TRUFLEX^®^ BL, SmartKen, Manchester, UK) of acrylate polymer was deposited onto the cleaned glass substrate via spin-coating, resulting in a crosslinked layer approximately 500 nm thick. Following this, a 50 nm thick gold (Au) layer was sputtered onto the BL, patterned through photolithography, and wet-etched to form the source and drain electrodes shown in Figure 1b. The source and drain electrodes were further modified using a self-assembled monolayer (TRUFLEX^®^ SAM) technique, applied onto the electrodes for 1 min, followed by spin-coating at 1000 rpm for 20 s. This was repeated with two cycles of IPA flooding and spin-coating to remove any excess SAM material, after which the substrate was baked at 100 °C for 1 min and allowed to cool to room temperature. Subsequently, the channel layer was fabricated by spin-coating a blended organic semiconductor (TRUFLEX^®^ OSC) solution, initially at 500 rpm for 10 s, followed by 1250 rpm for 60 s. The sample was immediately baked at 100 °C for 1 min to stabilize the OSC layer. The organic gate insulator (TRUFLEX^®^ OGI) consisted of two layers. The low-k dielectric first layer was spin-coated at 1500 rpm for 20 s and baked sequentially at 50 °C for 1 min and 100 °C for 1 min, producing a 150 nm thick layer. The second layer, a stress-release polymer dielectric (so-called TRUFLEX^®^ SRL), was deposited via spin-coating at 500 rpm for 10 s and 1250 rpm for 180 s. This layer underwent UV curing using a broadband mercury lamp (4200 mJ/cm^2^) under nitrogen flow, followed by baking at 120 °C for 5 min, achieving a thickness of about 400 nm. After forming the OGI, a 50 nm thick Au layer was sputtered and patterned to create the gate electrode shown in Figure 1c. Reactive ion etching (RIE) was used to pattern the OSC layer using the metal gate as a mask. A passivation layer (TRUFLEX^®^ PV) was then deposited, baked at 100 °C for 60 s, UV cured at 4200 mJ/cm^2^ under nitrogen flow, and baked again at 120 °C for 5 min. The PV, with a total thickness of 2 μm, was patterned with vias using photolithography and RIE etching to allow interconnections to the back-gate metal layer shown in Figure 1d. Finally, as shown in Figure 1e, a 50 nm Mo metal layer was sputtered and patterned to form the gate interconnect wiring and serve as a light-blocking layer [19], completing the fabrication process of the OTFT.

### 2.2. Mini-LED Die Bonding

**Figure 2:**
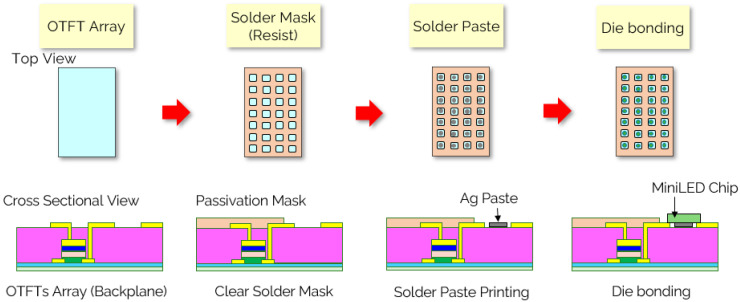
Process flow of mini-LED die bonding process.

In Figure 2, the process of mini-LED bonding using conductive silver paste (RS Pro Conductive Paint, RS Components Ltd., Northants, UK) involved several critical steps to ensure optimal electrical and mechanical stability. Initially, the substrate was prepared and cleaned to remove any contaminants that could affect adhesion. Conductive silver paste, consisting of finely dispersed silver particles in a polymer binder, was then precisely applied to the substrate using methods such as screen printing or dispensing. This facilitated the formation of intricate patterns essential for the dense arrangement of mini-LED arrays. Following the application, mini-LED chips were accurately placed onto the substrate, ensuring proper alignment and contact with the conductive silver paste. The assembly was subsequently subjected to a curing process, typically involving heat treatment, to solidify the paste, thereby ensuring strong electrical connections and mechanical stability. This method leveraged the excellent electrical conductivity and thermal stability of silver, making it ideal for fabricating high-performance mini-LED displays with enhanced brightness and reliability.

### 2.3. Powering up Mini-LED Backlights

The process of powering up (panel light-on) the mini-LED backlights involved the use of anisotropic conductive film (ACF) bonding followed by control and testing using a field-programmable gate array (FPGA). Several critical steps were taken to ensure effective electrical connections and functionality. Initially, the ACF, which contained conductive particles suspended in an adhesive matrix, was applied to the backplane substrate. The fan-outs from the FPC were then carefully aligned and placed onto the ACF-coated substrate. Under heat and pressure, the conductive particles within the ACF formed vertical (Z-axis) electrical connections between the flex film and the backplane circuits while the adhesive secured the components in place. Once the bonding process was complete, an FPGA was programmed to control and test the mini-LED array. The FPGA managed the lighting sequences, brightness levels, and display patterns of the mini-LED, enabling comprehensive testing and adjustment. By employing ACF bonding and FPGA control, this method provided a robust and flexible solution for developing and verifying the high-performance mini-LED backplanes.

### 2.4. Methods and Test Equipment

Electrical characteristics were evaluated in this study using an Agilent B1500A semiconductor device analyzer from Agilent Technologies (Santa Clara, CA, USA), manufactured in Hachioji, Japan. Measurements were conducted on a Cascade Microtech M150 platform produced by FormFactor (Livermore, CA, USA). Thermal imaging was performed using a TIM640 Thermo Imager, a compact infrared camera developed by MICRO-EPSILON (Ortenburg, Germany). Waveforms were recorded with a WAVESURFER 3054 oscilloscope from Teledyne LeCroy (Chestnut Ridge, NY, USA).

## 3. Results and Discussion

The specifications of the completed digital backlighting device, which integrates display backlight technology with active-matrix OTFTs, are detailed in this section.

### 3.1. Device Performance of 5-Masks OTFTs

**Figure 3:**
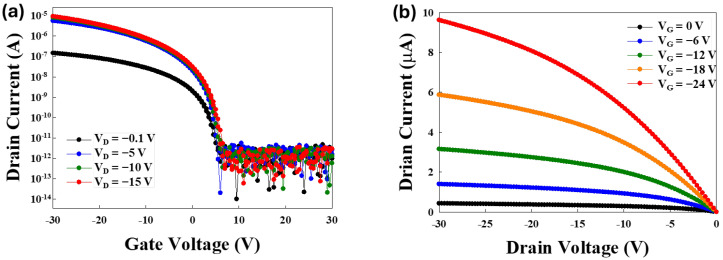
(**a**) *I_D_*-*V_G_* and (**b**) *I_D_*-*V_D_* characteristics of the OTFT.

The typical I-V characteristics of the OTFT device, as shown in Figure 3, include the transfer curve shown in Figure 3a and the output curve shown in Figure 3b. The device has a channel width (*W*) of 25 µm and a channel length (*L*) of 4 µm, with a gate capacitance per unit area (*C_ox_*) of 5 × 10^−9^ F/cm^2^. Using the linear region mobility formula shown as follows:

(1) $$\mu =\frac{L}{W\times C_{ox}\times V_{D}}{\times G}_{m}$$

at *V_D_* = −0.1 V, the calculated field-effect mobility (*μ*) was 2.15 cm^2^/V·s. At this condition, the transconductance (*G_m_*) was 5.21 × 10^−10^ S. The threshold voltage (*V_th_*) was determined using the equation:

(2) $$I_{D}=\mu \times C_{ox}\times \frac{W}{L}\times \left(V_{G}-V_{th}\right)\times V_{D}$$

The *V_th_* was determined to be 1.34 V when the drain current (*I_D_*) was 10^−9^ A. The subthreshold swing (SS) was calculated as:

(3) $$SS=\frac{dV_{G}}{\log(I_{D})}$$

based on the logarithmic relationship between *I_D_* and *V_G_* in the subthreshold region.

### 3.2. OTFTs Backplane Circuity

**Figure 4:**
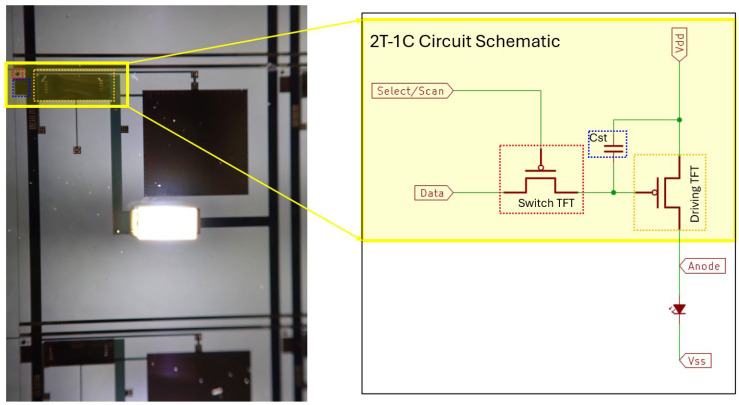
The 2T-1C circuitry consisted of two transistors and one capacitor, forming the core structure for precise brightness control in digital lighting applications.

The 2T-1C circuitry, comprising a driving TFT, a switching TFT, and a storage capacitor, was employed to construct an active-matrix driving scheme for enabling the digital lighting. As shown in Figure 4, the driving TFT was responsible for controlling the current supplied to the mini-LED, while the switching TFT enabled the pixel to be addressed through the V_scan_ signal (row). The storage capacitor maintained the voltage level applied to the driving TFT, ensuring stable operation during the frame period. The brightness of the LEDs was controlled by the V_data_ signal (column) in conjunction with the V_scan_ signal (row), which also served as the gamma voltage. The dynamic scanning input waveforms were carefully designed to achieve precise local dimming functionality, enhancing display contrast and reducing power consumption.

In addition, the active-matrix driving scheme minimized the need for direct connections to individual LEDs. Instead, driver IC connections were located only at the edges of the display, reducing the number of fan-outs compared to conventional LED lighting systems. This approach not only improved integration and scalability but also simplified the overall circuit design.

**Figure 5:**
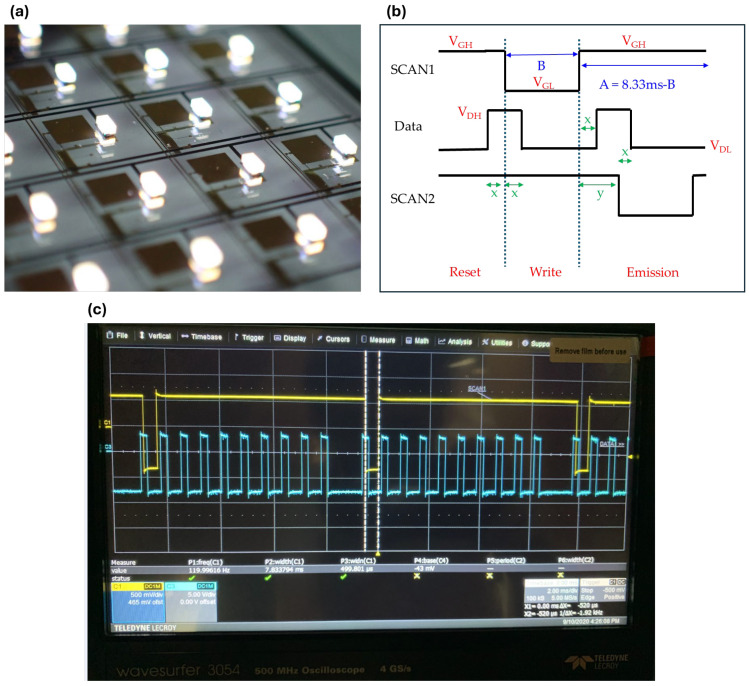
Overview of OTFT and mini-LED bonding and waveform implementations. (**a**) A visual image of mini-LEDs bonded on the OTFT backplane. (**b**) Designed driving waveform illustrating the operational stages of reset, write, and emission. The diagram highlights critical voltage levels (e.g., V_GH_, V_GL_, V_DL_) and timing intervals (e.g., A–B) required for AM operation. (**c**) Oscilloscope-measured waveforms of the AM operation. The left image presents the overall waveform, while the right images provide detailed views of the scan signal (top) and data signal (bottom), validating the designed driving mechanism through experimental measurements.

Figure 5 illustrates the operation of a mini-LED display driven by an OTFT backplane at a refresh rate of 120 Hz. In Figure 5a, a visual representation of the mini-LED bonded onto the OTFT backplane is shown. Figure 5b depicts the designed driving waveform, highlighting key operational stages: reset, write, and emission. The waveform includes critical voltage levels such as the gate low voltage (V_GL_: −8 V), gate high voltage (V_GH_: +6 V), output voltage drain driver (OV_DD_: +3 V), output voltage source supply (OV_SS_: −5 V), drain high voltage (V_DH_: 0 V), and drain low voltage (V_DL_: −6 V). Additionally, timing intervals essential for AM operation are specified, including A = 633 µs, B = 500 µs, x = 120 µs, and y = 250 µs. Figure 5c displays oscilloscope-measured waveforms, with the left image showing the overall waveform, while the right images provide detailed views of the scan signal (top) and data signal (bottom). These measurements validate the functionality and precision of the designed driving mechanism, ensuring the reliable performance of the mini-LED display system.

### 3.3. OTFTs Device Characteristics

**Figure 6:**
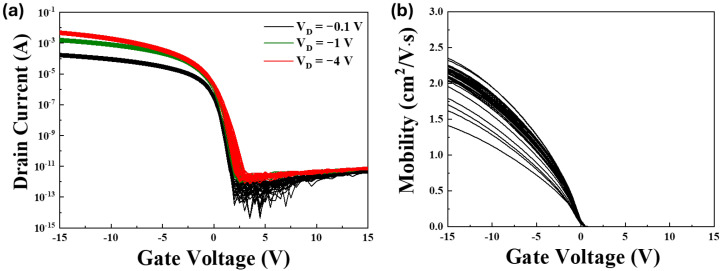
*I_D_*-*V_G_* characteristics of driving TFT devices. (**a**) The *I_D_-V_G_* characteristics of the driving TFT device were measured at varying V_data_ levels across 36 devices under three drain bias conditions (*V_D_*: −0.1 V, −1 V, −4 V). (**b**) The carrier mobility as a function of *V_G_* was calculated using the linear region formula with a *V_D_* of −0.1 V.

The driving TFTs with a channel width (*W*) of 110,000 µm, a channel length (*L*) of 4 µm, and *W*/*L* ratio of 27,500 characteristics are presented in Figure 6, which illustrates the electrical performance of the device under varying bias conditions. As shown in Figure 6a, the *I_D_*-*V_G_* curves demonstrate the relationship between *I_D_* and *V_G_* for three different drain voltages (*V_D_*): *V_D_* = −0.1 V, *V_D_* = −1 V, and *V_D_* = −4 V. These curves highlight the transistor’s turn-on behavior and subthreshold characteristics as the gate voltage varies. The observed positive *V_th_* and the increase in the drain current with decreasing *V_G_* suggest that the material exhibits p-type behavior, as the charge carriers are primarily holes.

This tendency is attributed to the inherent characteristics of the organic semiconductor material used in the OTFTs, which favors hole transport. Additionally, the sharper slope in the subthreshold region and the saturation of drain current at higher *V_G_* values indicate efficient charge injection and transport, as well as good device scalability.

In Figure 6b, the mobility—*V_G_* plot depicts the carrier mobility as a function of the gate voltage, calculated using the linear region formula with a *V_D_* of −0.1 V, shown in black color. The mobility increased with *V_G_*, suggesting enhanced charge accumulation at higher gate voltages, improving carrier transport. This trend further validates the p-type nature of the material and highlights the OTFT’s ability to achieve stable performance under varying gate bias conditions. The results indicate that the OTFTs delivered a drain current exceeding several milliamperes while maintaining an on/off ratio greater than 10 orders of magnitude. This performance underscores the suitability of the device for high-performance applications such as active-matrix mini-LED backlighting.

### 3.4. Lighting (Array Light-On) Features

**Figure 7:**
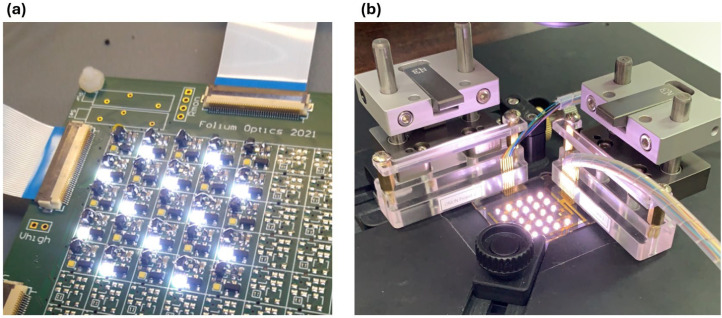
Comparison of Si-MOSFET circuit and AM OTFTs. (**a**) A 5 × 5 Si-MOSFET circuit with surface-mount capacitors, generated on a PCB using custom driver electronics. (**b**) AM OTFTs showcasing a simplified and cost-effective circuit design.

A 5 × 5 Si-MOSFET circuit with surface-mount capacitors, generated on a PCB using custom driver electronics, is shown on the left-hand side of Figure 7a, while the active-matrix OTFT circuit is displayed on the right. This comparison highlights that all MOS transistors, resistors, and capacitors were replaced by OTFT circuitry, significantly reducing integration complexity and cost. Figure 7b presents the output performance of the OTFT-based system, showing the checkerboard patterns used to validate the driving scheme. The uniformity of the brightness across the pattern confirms the effectiveness of the active-matrix OTFT in delivering consistent performance. Additionally, pulse width modulation (PWM) was employed to drive the LEDs, as illustrated in Figure 5b, effectively reducing heat dissipation and lowering power consumption [27]. These results demonstrate that the OTFT-based system not only achieves functional validation but also provides a scalable and efficient solution for mini-LED backlighting applications.

### 3.5. Performance of Digital Lighting

**Figure 8:**
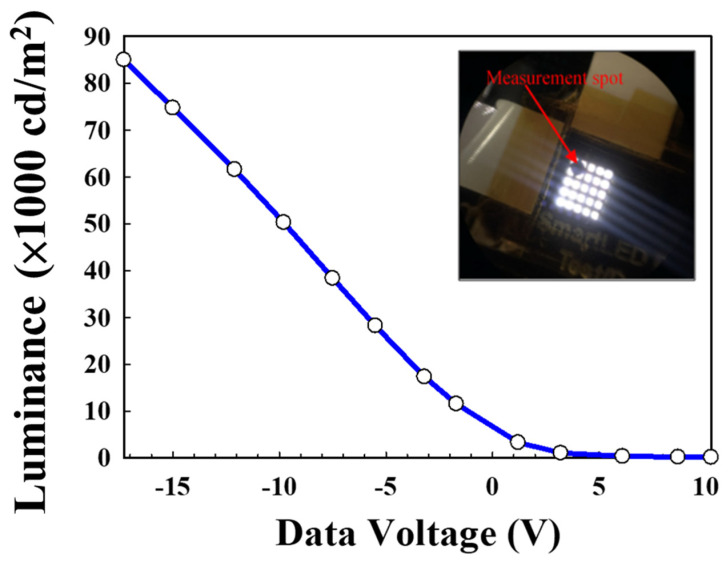
Performance of OTFT-driven digital lighting, with device luminance adjusted by different data signals and V_scan_ voltages.

During the digital lighting performance test, the panel demonstrated exceptional brightness, as shown in Figure 8. Under operating conditions of V_dd_ to V_ss_ = +8.55 V and V_select_ ranging from +24 V to −24 V, a luminance of 85,000 nits (cd/m^2^) was achieved, with an impressive contrast ratio of 1,000,000:1. Furthermore, the panel maintained a stable working temperature, with the LED temperature recorded at approximately 30 °C during operation.

### 3.6. Peak Brightness and Low-Temperature Driving

**Figure 9:**
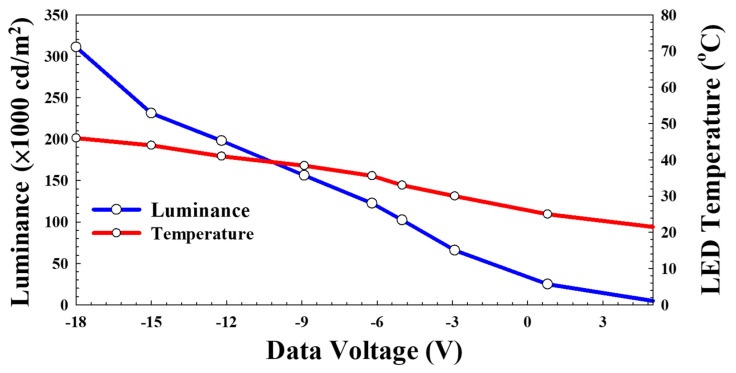
Monitoring the temperature of mini-LED devices while varying V_data_ to achieve high luminance.

When the V_data_ was adjusted to −18 V, the peak brightness reached an impressive 300,000 nits (cd/m^2^), as shown in Figure 9. Thanks to the active driving method, the actual operating temperature of the LEDs remained only slightly higher than room temperature. Compared to traditional passive-matrix-driven LEDs, active driving enables the use of lower voltages and allows for the integration of both PWM and DC dimming, enhancing efficiency and flexibility [28,29].

## 4. Conclusions

The use of OTFTs presents significant advantages, including flexibility, lightweight design, and compatibility with innovative display technologies. This work demonstrates the potential of OTFTs to drive digital mini-LED backlighting, achieving exceptional brightness levels exceeding 100,000 nits (cd/m^2^). The OTFTs exhibit excellent electrical performance, delivering milliampere-scale current while maintaining high on/off current ratios exceeding 10 orders of magnitude, stable threshold voltages, and efficient charge transport, as validated through comprehensive electrical characterization.

By integrating OTFTs with mini-LED technology, this study achieved superior contrast ratios of 1,000,000:1, enhanced energy efficiency, and improved color brightness. The active-matrix OTFT fabricated under a low-temperature process (below 120 °C) further ensures a stable substrate with effective heat management, improving the overall performance and reliability of the digital lighting system. These findings highlight the ability of OTFTs to meet the demands of next-generation display technologies, paving the way for advancements in energy-efficient and high-performance digital lighting applications. This study underscores the transformative potential of OTFTs in revolutionizing display and lighting systems, emphasizing the novelty and significance of this approach in addressing the challenges of modern display technology.

## Data Availability

The data presented in this study are available from the corresponding author upon reasonable request.
